# Has upwelling strengthened along worldwide coasts over 1982-2010?

**DOI:** 10.1038/srep10016

**Published:** 2015-05-08

**Authors:** R. Varela, I. Álvarez, F. Santos, M. deCastro, M. Gómez-Gesteira

**Affiliations:** 1 EPhysLab, Departamento de Física Aplicada, Facultade de Ciencias, Universidade de Vigo, Ourense, España; 2 CESAM, Departamento de Física, Universidade de Aveiro, 3810-193, Aveiro, Portugal

## Abstract

Changes in coastal upwelling strength have been widely studied since 1990 when Bakun proposed that global warming can induce the intensification of upwelling in coastal areas. Whether present wind trends support this hypothesis remains controversial, as results of previous studies seem to depend on the study area, the length of the time series, the season, and even the database used. In this study, temporal and spatial trends in the coastal upwelling regime worldwide were investigated during upwelling seasons from 1982 to 2010 using a single wind database (Climate Forecast System Reanalysis) with high spatial resolution (0.3°). Of the major upwelling systems, increasing trends were only observed in the coastal areas of Benguela, Peru, Canary, and northern California. A tendency for an increase in upwelling-favourable winds was also identified along several less studied regions, such as the western Australian and southern Caribbean coasts.

Wind-driven coastal upwelling results from the action of winds along a coast that generate an Ekman drift directed offshore. This causes pumping of cool and nutrient-rich water towards the sea surface along a narrow region close to the coast, which enhances primary production. Thus, coastal upwelling systems are among the most productive marine regions in the world’s oceans.

Due to the ecological and economic importance of these regions, changes in upwelling strength and timing have attracted considerable scientific interest in recent decades. In several studies, researchers have primarily focussed on analysing trends in wind strength due to climate variability and the resultant changes in the coastal upwelling process. In 1990, Bakun^1^ reported the strengthening of upwelling intensity along the major coastal upwelling systems of the world from 1945 to 1985. He proposed that this increase was due to global warming, which would create an intensification of the land-sea thermal contrast. This intensification would be reflected in increased land-sea pressure gradients, which in turn would cause the strengthening of upwelling-favourable winds that would result in cooling of the ocean surface.

Studies focused on different upwelling regions have been conducted to investigate the Bakun hypothesis through an analysis of available wind data. However, these studies reported contradictory results, which indicate that wind estimates from different databases can differ in trends and variability^2^. In addition, existing time-series data are limited in duration, quality, and spatial extent, and results obtained from different data products in the same area may vary because they are highly dependent on the length of the time series. Recently, Sydeman *et al.*^3^ conducted an analysis of the literature on upwelling-favourable winds along the major eastern boundary current systems to test the Bakun hypothesis. They synthesized results from more than 20 studies published between 1990 and 2012 based on time series ranging in duration from 17 to 61 years. Most of published data support general wind intensification in the California, Benguela, and Humboldt upwelling systems and weakening in the Iberian system. This study highlighted the dependence of the results on the length of the time series and season, and it revealed contradictory results between observational data and model-data reanalysis. In addition, numerous available time series present a spatial resolution that is too coarse to accurately resolve conditions at the scale of coastal upwelling in intense and localized upwelling zones. Thus, higher-resolution temporal scales and greater spatial-resolution studies are needed.

The aim of this study was to identify the temporal and spatial trends in coastal upwelling regimes worldwide using wind stress data from the National Centers for Environmental Prediction (NCEP) Climate Forecast System Reanalysis (CFSR)^4^ database. This database provides high spatial resolution (approximately 0.3°) with data available from 1982 to 2010. The length of this database allows detailed estimation of upwelling trends over the recent period of strong global warming, and the same database can be used for all areas of interest.

## Methods

Wind data were acquired from the NCEP CFSR database at http://rda.ucar.edu/pub/cfsr.html developed by the National Oceanic and Atmospheric Administration (NOAA). Data were retrieved from the NOAA National Operational Model Archive and Distribution System, which is maintained by the NOAA National Climatic Data Center. Detailed information about the CFSR database can be found in Saha *et al.*^4^. This worldwide database has a spatial resolution of approximately 0.3 × 0.3° and a temporal resolution of 6 hours from January 1982 to December 2010. The reference height of the wind data is 10 m. Coastal upwelling analysis requires the use of pixels as close to shore as possible to represent coastal processes. To avoid land contamination, only coastal pixels with less than 25% of land were used.

The process used to identify upwelling areas and to calculate trends is summarized below:

1. Wind data were initially averaged at a daily scale to smooth the effect of high frequency events such as breezes. In addition, upwelling events typically last 3–14 days^5^, so a daily scale seems accurate to describe them.
2. Alongshore wind stress 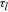 was calculated using the equation 
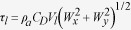
, where 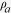 is the air density, 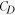 is the drag coefficient, and 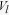 is the upwelling-favourable wind. 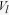 can be calculated as 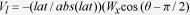
+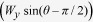, where 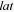 is the latitude, 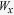 is the zonal wind component, 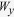 is the meridional wind component, 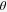 is the angle defined by an unitary vector normal to the shoreline and pointing seaward and *abs* means absolute value. Alongshore wind stress has been previously used to estimate variations in upwelling intensity^1^,^6^,^7^. This variable provides information about upwelling intensity spreading both in time and in space without gaps, even in regions close to the Equator were other variables such as Ekman transport diverge. 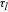 and 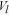 were stored at the daily scale for each coastal pixel over the period 1982–2010.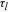 and 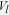 were averaged at a monthly scale to obtain 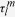 and 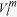 because the study was not focused on particular events but rather on identifying regions that show well-developed upwelling conditions lasting for long periods.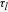 and 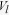 climatology (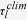 and 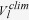) was calculated for the whole period. Different conditions were imposed on these variables to identify upwelling areas. First, 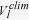 must be higher than 5.4 ms^–1^, which corresponds to the transition from a gentle to a moderate breeze on the Beaufort scale. García-Reyes *et al.*^5^ defined upwelling events as periods of time with alongshore winds stronger 5 ms^–1^ following Cury and Roy^8^. Sensibility tests have shown that results are independent of the particular value of the threshold within the range 5 to 5.5 ms^–1^. This condition must be fulfilled for at least three consecutive months. Second, the area is considered to be an upwelling region only if at least 10 consecutive points (about three degrees) fulfil the previous condition. This second condition discards the appearance of small local areas.
5. Trends were calculated using only the months under strong upwelling conditions. Those months were selected from the climatology data considering 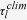 values higher than the 50% percentile. As a consequence, the number of months per year to be used in the analysis differed from zone to zone. This number varied from five to seven months, dependent on the area, with six months being the most consistently found value.
6. Trends were calculated at each pixel as the slope of the linear regression of the alongshore monthly wind stress anomalies versus time. Monthly anomalies were calculated by subtracting from the alongshore wind stress of a certain month (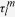) the mean alongshore wind stress of that month over the period 1982–2010^9^,^10^. All trends were calculated using raw data without any filter or running mean. The Spearman rank correlation coefficient was used to analyse the significance of trends due to its robustness to deviations from linearity and its resistance to the influence of outliers^9^,^10^. The significance level of each pixel is shown in the figures for those points that exceed 90% (circle) or 95% (square) of significance.
7. All figures herein were generated using Matlab.

## Results and discussion

The present study investigated trends in coastal upwelling for coastal regions of the world using the CFSR wind database with high spatial resolution (approximately 0.3°). This database facilitates the analysis of wind behaviour at a small scale, which is a key factor when considering coastal mesoscale effects as upwelling. Recent studies have compared this database and different wind products with wind measured by several buoys along the Iberian Peninsula coast^11^,^12^,^13^. Statistical results confirmed that datasets with finer spatial resolution, such as CFSR, gave better results, especially near the coast.

In this study, 3025 coastal points worldwide were analysed to select the major upwelling regions and evaluate upwelling trends from 1982 to 2010. Under our terms of selection, ten upwelling systems were evaluated: Benguela, Canary, the southern Caribbean Sea, Chile, Peru, California (north and south), West Australia, Java, North Kenya, and Somalia–Oman. These systems were grouped together into several macroscopic zones, namely the eastern and western coasts of the Atlantic, Pacific, and Indian Oceans. No upwelling systems along the western Pacific Ocean were assessed. Alongshore wind stress was calculated over more than 600 points along this coast, and no region fulfilled the conditions required to be considered a coastal upwelling area.

### Eastern Atlantic Ocean

**Figure 1:**
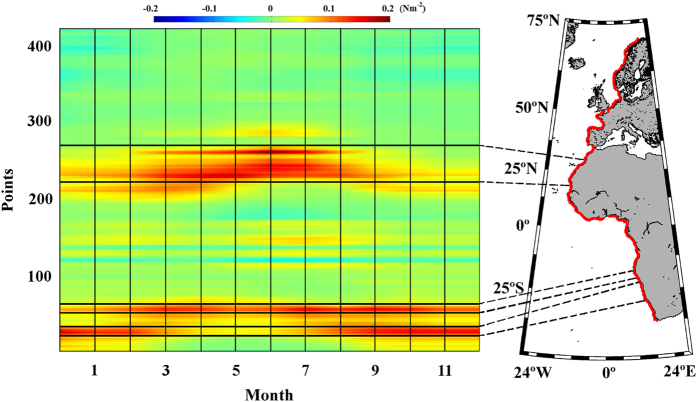
Wind stress along the Eastern Atlantic Ocean. Annual cycle of alongshore wind stress (Nm^–2^) for the period 1982–2010. Red points on the map show the coastal points studied. Black lines indicate those regions considered to be coastal upwelling areas. This figure has been performed using Matlab.

Two upwelling systems were analysed along this coast from south to north: Benguela (South 30.13–23.26°S; North 20.76–16.39°S) and Canary (18.89–32.63°N) (Fig. 1). Along the Benguela upwelling system, which is one of the major upwelling regions of the world, two different areas (south and north) were assessed based on the results of the calculated alongshore wind stress (Fig. 1). Previous studies were focused mainly on the southern area from the southern tip of Africa to about 20°S. Nevertheless, several studies evaluated the entire area from south of 15°S. The results of the present study for the southern and northern areas will be described below, and then a comparison with results from previous studies will be presented.

### Benguela

**Figure 2:**
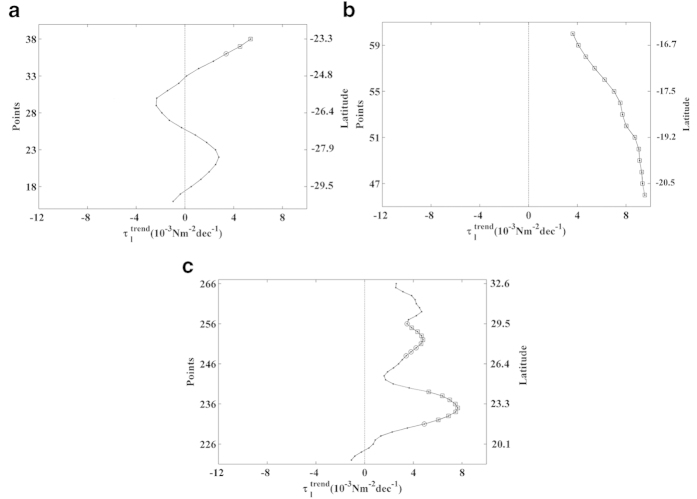
Upwelling trends in the selected areas along the Eastern Atlantic Ocean. (**a**) Alongshore wind stress trends along the southern Benguela coast calculated from September to March. (**b**) Alongshore wind stress trends along the northern Benguela coast calculated from July to November. (**c**) Alongshore wind stress trends along the Canary coast calculated from April to September. Those points with significance greater than 90% are marked with a circle, and those greater than 95% are marked with a square. A negative (positive) trend means a decrease (increase) in upwelling-favourable winds. This figure has been performed using Matlab.

**Figure 3:**
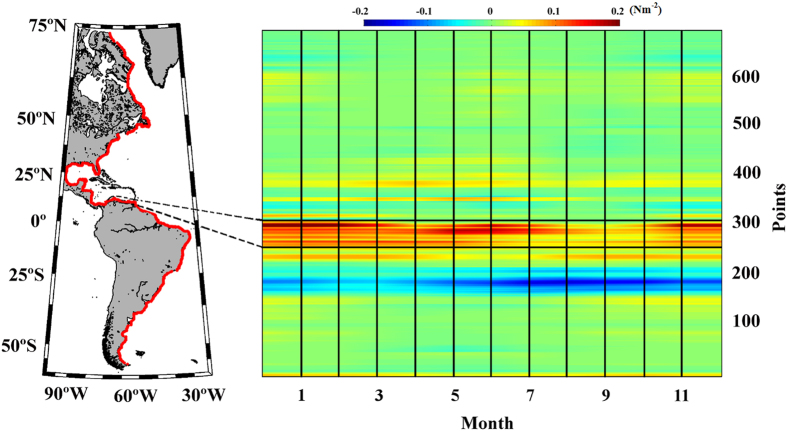
Wind stress along the Western Atlantic Ocean. Annual cycle of alongshore wind stress (Nm^–2^) for the period 1982–2010. Red points on the map show the coastal points analysed. Black lines indicate those regions considered to be coastal upwelling areas. This figure has been performed using Matlab.

At the southern coast of the Benguela system, alongshore wind stress had positive values (upwelling-favourable conditions) throughout the year, but the highest values occurred during the austral spring and summer months (Fig. 1, points 16–38). The upwelling season was defined as September to March based on the annual cycle of alongshore wind stress meridionally averaged over these points. 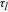 trends were calculated during the upwelling season (Fig. 2a). Non-significant trends were identified south of 24 °S, and only the three northernmost grid boxes (–23.3 to 24°S) displayed a significant positive trend. These significant positive trends continued throughout the Northern Benguela Zone (Fig. 3), although the defined upwelling season differed for this zone (from July to November). A significant positive trend was observed for the entire region, with values ranging from 4 10^−3^ Nm^–2 ^dec^–1^ in the northern area to 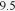 10^−3^ Nm^–2 ^dec^–1^ in the southern one.

Previous studies reported similar results. For example, Patti *et al.*^14^ analysed data from the Comprehensive Ocean-Atmosphere Data Set (COADS) and found an increase in annual wind stress (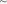
10 10^−3^ Nm^–2^ dec^–1^) for the area extending from 20 to 30°S, indicating upwelling reinforcement from 1958 to 2007. Narayan *et al.*^15^ described a significant increase (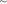
5 10^−3^ Nm^–2^ dec^–1^) in the COADS and National Center for Environmental Prediction/National Center for Atmospheric Research (NCEP/NCAR Reanalysis) meridional wind stress across 26–36°S from 1960 to 2001 using annual data. On the other hand, annual wind stress data from European Center for Medium range Weather Forecasting (ECMWF) Re-Analysis (ERA-40 Reanalysis) used in the same study^15^ showed a non-significant trend for the same period. Analysis of upwelling trends along the Benguela Upwelling Ecosystem was also conducted using a derived upwelling index in terms of Ekman transport to represent the estimated potential effects of wind stress on the ocean surface^9^,^16^. Pardo *et al.*^16^ and Santos *et al.*^9^ found a general increase in upwelling intensity over the last four decades for the areas 20–32°S and 16–30°S, respectively, using the NCEP/NCAR Reanalysis annual data.

### Canary

Along the Canary coast, positive values of alongshore wind stress (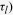 were observed throughout the year, with the highest values occurring in spring and summer months (Fig. 1, points 222–266). The upwelling season was considered to be April to September. 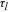 trends over this period were calculated (Fig. 2c), and positive trends were detected over almost the entire region. However, significant values were found only around 22–24°N (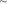
8 10^−3^ Nm^–2^ dec^–1^) and 27–29°N (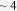 10^−3^ Nm^–2^ dec^–1^). Published results regarding trends in upwelling along the Canary coast are controversial. Trends in upwelling can be highly dependent on the length of the time series, the selected area, and the season evaluated in the analysis. Results similar to those found in the present study were reported by Cropper *et al.*^17^ using meridional wind speed from CFSR over the period 1981–2012 during summer (June-August). These authors found a non-significant increase in upwelling-favourable winds off Northwest Africa (11–35°N). On the other hand, different wind databases such as NCEP/Department Of Energy (NCEP/DOE II), ERA-Interim, 20 Century, and National Aeronautics and Space Administration-Modern Era Retrospective-Analysis for Research and Applications (NASA-MERRA) used in the same study for the same period showed a statistically significant increase north of 21°N and a generally significant decrease in upwelling-favourable winds south of 19°N. Results of previous studies are also in agreement with our finding of increasing trends in upwelling along the Canary coast. For example, McGregor *et al.*^18^ described increasing trends in upwelling around 31°N using annual wind stress data from COADS for the period 1950–1992. Narayan *et al.*^15^ and Patti *et al.*^14^ also found significant increasing trends (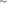
4-5 10^−3^ Nm^–2^ dec^–1^) using the same database across 24–32°N considering annual wind stress over the last four decades. Narayan *et al.*^15^ reported a significant increase (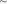
2 10^−3^ Nm^–2^ dec^–1^) in the ERA-40 Reanalysis meridional wind stress for the same period. In contrast, when they used the NCEP/NCAR reanalysis these authors^15^ identified a reduction in meridional wind stress (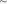
–4 10^−3^ Nm^–2^ dec^–1^) over the last four decades (1960–2001) in the same region (24–32 °N), indicating a reduction in coastal upwelling. More recently, Barton *et al.*^2^ conducted an extensive study of wind-induced upwelling trends along the whole Canary current upwelling system. Using monthly meridional wind data from the Pacific Fisheries Environmental Laboratory (PFEL), NCEP/NCAR, ECMWF, ICOADS, and Wave and Anemometer-based Sea Surface Wind (WASWind) plus data from coastal meteorological stations over 40 years (1967–2007), these authors found that trends varied among different data products in the same area, as both negative and positive trends were evident. In addition, not statistically significant changes in meridional wind components were found. Contradictory results were also obtained using Ekman transport data in numerous upwelling trend studies conducted along the Canary Upwelling Ecosystem. Gomez-Gesteira *et al.*^19^ detected a significant decreasing trend in upwelling strength for all seasons across 20–32°N from 1967 to 2006 using data from the PFEL. Pardo *et al.*^16^ also found a general weakening of the upwelling intensity along the Iberian/Canary (26–43°N) and NW African (10–24°N) regions from 1970 to 2009 using the NCEP/NCAR Reanalysis. These trends were clearly observed in winter and autumn for both regions, and a weakening in the upwelling intensity was also detected in summer in the northwest African region. In contrast, Santos *et al.*^10^ confirmed a spring-summer increasing trend across 22–33°N when they used the same database (NCEP/NCAR) from 1982 to 2010 in accordance with the present study. Opposite results observed in some of the studies described above using the same database (NCEP/NCAR) emphasize that linear trends are strongly dependent on the length of the time series and the season evaluated^2^,^20^.

### Western Atlantic Ocean

**Figure 4:**
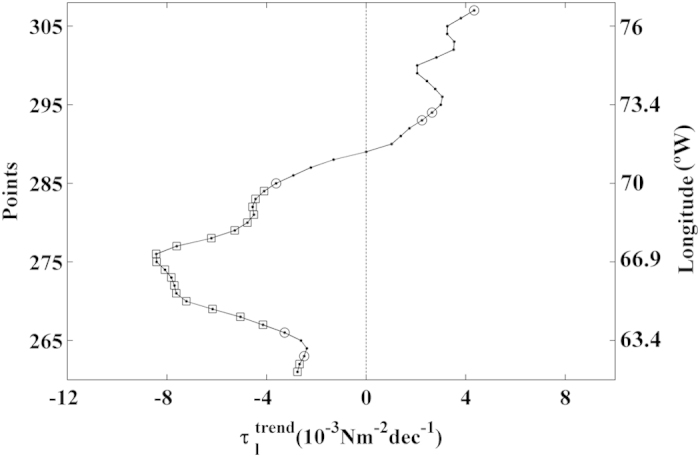
Upwelling trends in the selected areas along the Western Atlantic Ocean. Alongshore wind stress trends in the southern Caribbean Sea calculated from December to April. Those points with significance greater than 90% are marked with a circle, and those greater than 95% are marked with a square. A negative (positive) trend means a decrease (increase) in upwelling-favourable winds. This figure has been performed using Matlab.

Only one region along the coast of the western Atlantic Ocean fits the conditions required to be considered an coastal upwelling area: the southern Caribbean Sea (62.19–76.56°W) (Fig. 3). In the southern Caribbean upwelling region, alongshore wind stress had positive values throughout the year, with the highest values during the boreal winter months (Fig. 3, points 261–307). The upwelling season was considered to occur from December to April, and 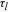 trends were calculated for this period (Fig. 4). The eastern and western regions exhibited different behaviours. A negative trend was detected east of 71.25°W, with a significance level higher than 90% (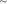
– 4 10^−3^ to – 8 10^−3^ Nm^–2^ dec^–1^) for almost all points. In the western region (71.25–76.56 °W) a non-significant positive trend was observed in most of the area, with maximum values of around 4 10^−3^ Nm^–2^ dec^–1^. This region was previously studied mainly in terms of upwelling occurrence using wind and sea surface temperature data^21^,^22^,^23^,^24^,^25^. As far as we know, no studies of upwelling trends in terms of wind have been conducted along the southern Caribbean upwelling system.

### Eastern Pacific Ocean

**Figure 5:**
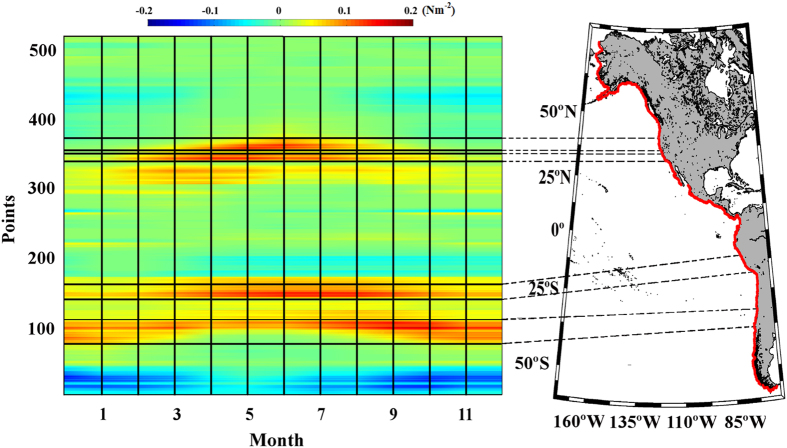
Wind stress along the Eastern Pacific Ocean. Annual cycle of alongshore wind stress (Nm^–2^) for the period 1982–2010. Red points on the map show the coastal points analysed. Black lines indicate those regions considered to be coastal upwelling areas. This figure has been performed using Matlab.

Three upwelling systems were evaluated along this coast from south to north: Chile (37.62–28.88 °S), Peru (16.39–10.15 °S), and California (South 33.88–36.06 °N; North 37.94–42. 31 °N) (Fig. 5).

### Chile

**Figure 6:**
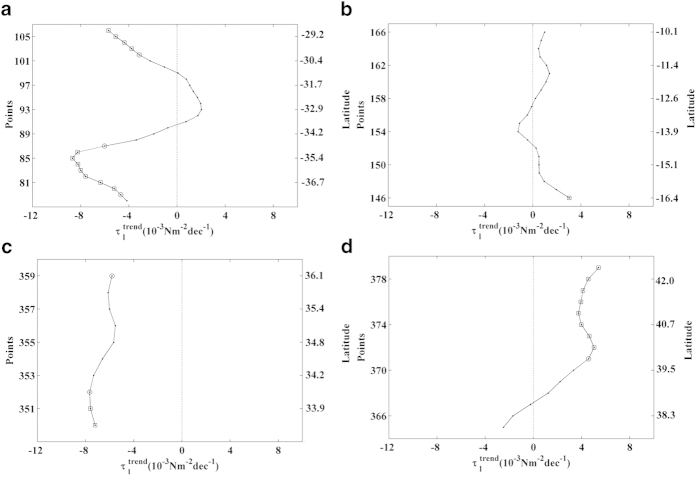
Upwelling trends in the selected areas along the Eastern Pacific Ocean. (**a**) Alongshore wind stress trends along the Chile coast calculated from October to April. (**b**) Alongshore wind stress trends along the Peru coast calculated from May to October. (**c**) Alongshore wind stress trends along the southern California coast calculated from April to September. (**d**) Alongshore wind stress trends along the northern California coast calculated from April to October. Those points with significance greater than 90% are marked with a circle, and those greater than 95% are marked with a square. A negative (positive) trend means a decrease (increase) in upwelling-favourable winds. This figure has been performed using Matlab.

Along the coast of Chile, positive values of alongshore wind stress were observed throughout the year, with the highest values during the austral spring and summer months (Fig. 5, points 78–106). The upwelling season was considered to last from October to April, and 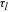 trends were calculated for these months (Fig. 6a). Significant negative trends were observed south of 34 °S and north of 31 °S, with values between –4 10^−3^ and –8 10^−3^ Nm^–2^ dec^–1^. Non-significant positive trends were detected at midlatitudes. Previous studies along this coast have reported different results. For example, Garreaud and Falvey^26^ analysed changes in the coastal winds along the west coast of subtropical South America (20–55°S; 70–85°W) using future climate scenarios. Different simulations were performed using the Providing REgional Climate for Impact Studies (PRECIS) regional climate model, and a significant trend in the coastal wind was absent during the late twentieth century (1961–1990). Similar results were observed by Goubanova *et al.*^27^, who used a statistical downscaling method to refine the representations of coastal winds for a global-coupled general circulation model (Institut Pierre Simon Laplace Climate Model (IPSL-CM4)) from 1970 to 1999. More recently, Rahn and Garreaud^28^ used CFSR over the period 1979–2010 to present a synoptic climatology of the coastal wind along the Chile/Peru coast, paying special attention to prominent upwelling regions. Points located along the Chile coast (30 and 36.4°S) showed unclear trend over the last 30 years. Finally, Aravena *et al.*^29^ used anomalies of Ekman transport data from 1980 to 2010 to assess the interannual evolution of upwelling along the northern-central coast of Chile (29–34°S). These Ekman transport anomalies obtained from PFEL showed a positive trend throughout the area. As previously mentioned, trends in upwelling can be highly dependent on factors such as the season evaluated in the analysis. This could explain the observed weakening in upwelling in most of the region in the present study, which we calculated using alongshore wind stress from October to April. When we recalculated 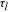 trends using annual data, the observed trend was unclear, which is in accordance with most of the studies previously conducted along this upwelling system. The dependence of trends on the season studied was also reported by Sydeman *et al.*^3^, who analysed more than 20 studies related to upwelling trends along the five major upwelling systems published over the last two decades. They found that some of the disagreement in previous studies could be resolved by considering winds during only the active upwelling seasons.

### Peru

Along the Peru coast, alongshore winds had positive values throughout the year, with maxima from May to October (Fig. 5, points 146–166). The upwelling season was considered to occur during these months, and 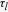 trends were calculated for this time period (Fig. 6b). Small and non-significant positive trends were observed for almost the entire region except at the southernmost point (16.4°S), where a significance level higher than 95% was identified (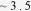 10^−3^ Nm^–2^ dec^–1^). A non-significant negative trend with small values was observed at 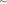
14°S. Previous studies have shown that trends in upwelling along the Peru coast are contradictory. Results similar to those obtained in our study were reported by Rahn and Garreaud^28^, who analysed annual alongshore winds from CFSR from 1979 to 2010. These authors found a positive trend in alongshore wind from 1995 to 2010 (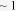 ms^–1^) at 15°S, which is a prominent upwelling region along the Peru coast. Gutierrez *et al.*^30^ used ERA-40 Reanalysis in a region around 14°S and also found an increase in upwelling-favourable winds from 1958 to 2001 for the spring season and using annual data. Bakun *et al.*^31^ reported an increase in upwelling at ~10.5°S using monthly wind stress data from COADS from 1948 to 2006. In contrast, Goubanova *et al.*^27^ suggested the existence of a weakening of alongshore wind near 15°S from 1970 to 1999 based on statistical downscaling of the sea surface wind. Previous studies conducted over a wider region have also shown different results. Patti *et al.*^14^ described an increase in annual wind stress using data from COADS for the area extending from 6 to 16°S and demonstrated the existence of upwelling reinforcement from 1958 to 2007. However, the trend they reported (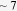 10^−3^ Nm^–2^ dec^–1^) was much higher than that found in the present study. Narayan *et al.*^15^ also analysed the same region from 1960 to 2001 using different datasets. Annual wind stress data from COADS (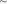
5 10^−3^ Nm^–2^ dec^–1^) and ERA-40 Reanalysis (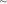
0.9 10^−3^ Nm^–2^ dec^–1^) revealed a significant increasing trend in upwelling, in agreement with the results reported by Patti *et al.*^14^, although in the latter case the trend value was much smaller. The general trend^14^,^15^,^31^ is a similar to that observed in the present study. In contrast, Narayan *et al.*^15^ identified a statistically non-significant decrease (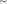
–0.7 10^−3^ Nm^–2^ dec^–1^) at the Peruvian upwelling region (6–16°S) using meridional wind stress from the NCEP/NCAR reanalysis. Pardo *et al.*^16^ analysed annual Ekman transport using data from the NCEP/NCAR Reanalysis and found a general weakening of the upwelling intensity in the Peru region (6.7–16.2°S) from 1970 to 2009.

### California

For the California upwelling region (Fig. 5), two different areas were assessed: South (33.88–36.06 °N) and North (37.94–42.31 °N). Almost all published reports about trends in upwelling along this system include the entire coast of California (32–42 °N). The results of the present study for the southern and northern areas will be described below, and then a comparison with results from previous studies will be presented.

Along the southern California coast, the highest values of alongshore wind stress were observed during the spring and summer months (Fig. 5, points 350–359). The upwelling season was considered to occur from April to September based on the annual cycle of wind stress meridionally averaged over these points, and 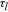 trends were calculated (Fig. 6c). Negative trends were observed for the entire region, but they were significant (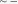
8 10^−3^ Nm^–2^ dec^–1^) only at the three southernmost points and at the northernmost one. Along the northern California coast, alongshore wind stress showed a similar behaviour, although the highest values were mainly observed in summer (Fig. 5, points 365–379). Thus, 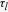 trends were calculated between April and October (Fig. 6d). Non-significant negative trends were detected for the three southernmost points, with maximum values around –3 10^−3^ Nm^–2^ dec^–1^. Positive trends were observed north of 38.5 °N with a significance level higher than 90% for the northernmost region (4-6 10^−3^ Nm^–2^ dec^–1^).

Controversial results in relation to the long-term variability in coastal upwelling were also found in the California upwelling system. In terms of wind speed, Mendelssohn and Schwing^32^ reported trends of stronger upwelling-favourable winds along 32–40°N based on April–September COADS data from 1946 to 1990. Patti *et al.*^14^ analysed a similar area between 34 and 40°N using annual wind stress data from the same database over the period 1958 to 2007. They described an increasing trend of around 4 10^−3^ Nm^–2^ dec^–1^. Narayan *et al.*^15^ also found a statistically significant increase in upwelling-favourable winds (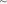
3 10^−3^ Nm^–2^ dec^–1^) using annual wind stress data from the same dataset from 1960 to 2001 for the same region. Although the southern area evaluated in the present study (33.88–36.06°N) is included in the region studied by Mendelssohn and Schwing^32^, Patti *et al.*^14^, and Narayan *et al.*^15^, the general trend observed in these three works and the present one is contradictory. In contrast, Narayan *et al.*^15^ identified a significant decreasing trend in upwelling from the ERA-40 Reanalysis of annual wind stress data (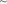
–0.6 10^−3^ Nm^–2^ dec^–1^) from 34 to 40°N over the last four decades (1960–2001). This decrease is in good agreement with the results of the present study, although the trend value was much higher in our case (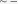
8 10^−3^ Nm^–2^ dec^–1^). Different results were also reported in several studies that covered a wider region. For example, Garcia-Reyes and Largier^33^ studied the California region from 33 to 42°N from 1982 to 2008 using wind speed data during the upwelling season (March–July) from the National Data Buoy Center (NDBC) buoys. They found significant increasing trends in upwelling winds north of 35°N and a decreasing trend in the southern region (33–35°N). Similar results were observed when data from the NDBC for June-August over the period 1980 to 2010^34^ were used. The decreasing trend along the southern coast of California (33–35°N) reported in these two works is in agreement with the results of our study. On the other hand, positive trends were only observed north of 38.5°N in our case.

Ekman transport data also have been evaluated in different studies conducted along the California Upwelling Ecosystem. Rykaczewski and Checkley^35^ found a positive summer trend around 34.5°N for the period 1948–2004 using Ekman transport data from the California Reanalysis Downscaling (CaRD10), which is a dynamically downscaled analysis of the NCEP/NCAR Reanalysis. Seo *et al.*^34^ found similar results using the same database over the entire California coast (32–42°N) from 1980 to 2010. Garcia-Reyes and Largier^33^ detected significant increasing trends in upwelling strength during March-July north of 34.5°N from 1982 to 2008 using data from the PFEL. Pardo *et al.*^16^ also studied the California region from 33 to 45°N using NCEP/NCAR Reanalysis Ekman transport data from 1970 to 2009, and they reported an unclear annual trend. In contrast, Iles *et al.*^36^ found an increasing trend in Ekman transport annual data from PFEL from 1967 to 2010 over the same region.

Considering that ENSO (www.esrl.noaa.gov) can be an important source of variability along the Pacific upwelling systems, its influence on the estimated trends of 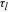 was analysed. No correlations were found between the variability of ENSO and 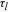.

### Eastern Indian Ocean

**Figure 7:**
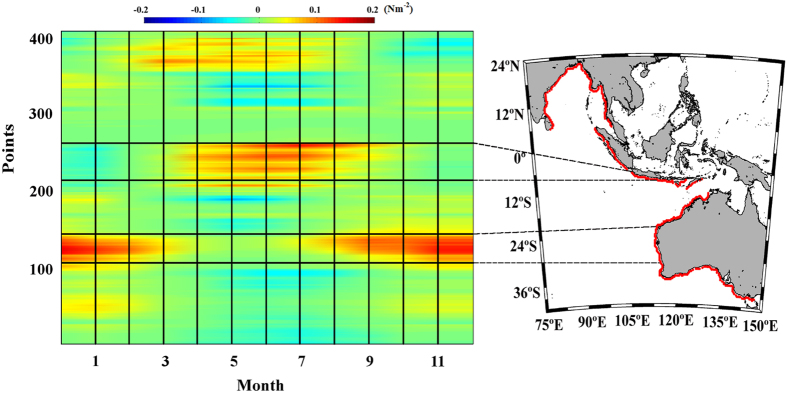
Wind stress along the Eastern Indian Ocean. Annual cycle of alongshore wind stress (Nm^–2^) for the period 1982–2010. Red points on the map show the coastal points analysed. Black lines indicate those regions considered to be coastal upwelling areas. This figure has been performed using Matlab.

Two upwelling systems were assessed along this coast: West Australia (31.69–21.39 °S) and Java (105.62–116.87 °E) (Fig. 7).

### West Australia

**Figure 8:**
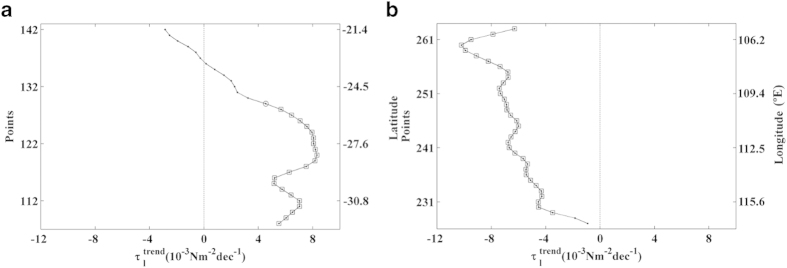
Upwelling trends in the selected areas along the Eastern Indian Ocean. (**a**) Alongshore wind stress trends along the Western Australia coast calculated from October to March. (**b**) Alongshore wind stress trends along the Java coast calculated from May to October. Those points with significance greater than 90% are marked with a circle, and those greater than 95% are marked with a square. A negative (positive) trend means a decrease (increase) in upwelling-favourable winds.

Along the western Australian coast, maximum values of alongshore wind stress were observed from October to March, which corresponds to the austral spring and summer months (Fig. 7, points 108–142). The upwelling season was considered to occur from October to March, and 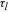 trends were calculated over these months (Fig. 8a). Positive trends were detected for almost the whole region, with significant values between 4-8 10^−3^ Nm^–2^ dec^–1^ south of 25.5°S. Non-significant negative trends were found at the northernmost latitudes (21.4–22.9°S). As far as we know, no studies regarding upwelling trends in terms of wind have been conducted along the western coast of Australia. Previous studies have shown the absence of persistent upwelling off this coast despite a prevailing summer wind system favouring upwelling. This absence of upwelling has been attributed to the presence of the Leewin Current (LC), a warm poleward flow transporting nutrient-poor waters from the tropics^37^,^38^. Unlike other eastern boundary currents (e.g., the Benguela and Humboldt Currents at similar latitudes), the poleward flow of the LC suppresses the persistent upwelling of cool, nutrient-rich, subsurface water onto the western Australia continental shelf^39^,^40^. Thus, large-scale upwelling is incompatible with the poleward flowing LC. Nevertheless, localized seasonal upwelling associated with inner shelf wind-driven currents can appear along some regions, such as the Ningaloo (23–25°S) and Capes Currents (26–28°S), due to variations in the LC^41^,^42^,^43^,^44^,^45^,^46^,^47^.

### Java

Along the Java coast, values of alongshore wind stress were higher during the austral winter (Fig. 8, points 227–263). The upwelling season was considered to last from May to October, and 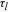 trends were calculated for this time period (Fig. 8b). Significant negative trends were detected for almost the entire coast, with values between –4 10^−3^ Nm^–2^ dec^–1^ at the easternmost region and –10 10^−3^ Nm^–2^ dec^–1^ at the westernmost one. The existence of upwelling along the Java coast and its basic features have been documented in previous studies, mainly in terms of upwelling occurrence determined using wind and SST data, whereas upwelling trends have not been considered. Different researchers have found that upwelling occurs between June and November and is mostly forced both locally by the alongshore winds associated with the southeast monsoon and remotely by atmosphere-ocean circulation associated with ENSO^48^,^49^,^50^,^51^,^52^,^53^.

### Western Indian Ocean

**Figure 9:**
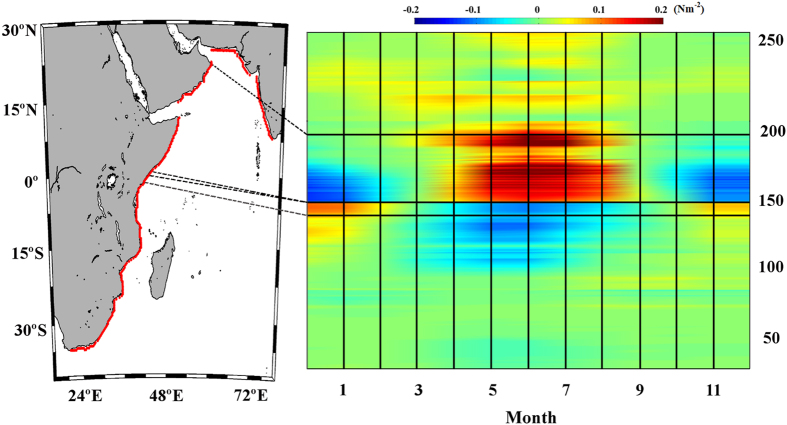
Wind stress along the Western Indian Ocean. Annual cycle of alongshore wind stress (Nm^–2^) for the period 1982–2010. Red points on the map show the coastal points analysed. Black lines indicate those regions considered to be coastal upwelling areas. This figure has been performed using Matlab.

Two upwelling systems were evaluated along this coast from south to north: North Kenya (2.03–1.40°N) and Somalia-Oman (1.72–22.01°N) (Fig. 9).

### North Kenya

**Figure 10:**
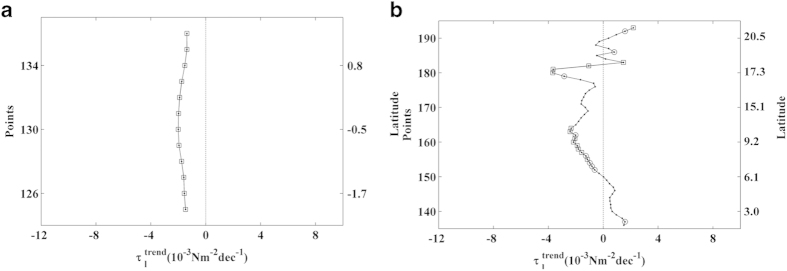
Upwelling trends in the selected areas along the Western Indian Ocean. (**a**) Alongshore wind stress trends along the Northern Kenya coast calculated from November to April. (**b**) Alongshore wind stress trends along the Somalia-Oman coast calculated from April to October. Those points with significance greater than 90% are marked with a circle, and those greater than 95% are marked with a square. A negative (positive) trend means a decrease (increase) in upwelling-favourable winds. This figure has been performed using Matlab.

Along the northern Kenya coast, positive values of alongshore wind stress were observed from November to April (Fig. 9, points 125–136). The upwelling season was considered to occur from November to April, and 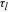 trends were calculated over this period (Fig. 10a). Significant negative trends were detected for the entire region, with values of around –2 10^−3^ Nm^–2^ dec^–1^. The coast of northern Kenya is characterized by the occurrence of an irregular upwelling linked to the northeast monsoon, which normally develops from November to March. Several researchers have related the occurrence of this upwelling to higher productivity in the area, although the fact that upwelling events are not regular has attracted relatively little scientific interest about upwelling trends in terms of wind. This region has been studied mainly in terms of changes in chemical and biological oceanographic parameters related to the occurrence of the northeast and southeast monsoons, which lead to important differences between the Kenya and Somalia coasts in terms of upwelling^54^,^55^,^56^,^57^,^58^,^59^,^60^.

### Somalia-Oman

Along the Somalia-Oman coast, positive values of alongshore wind stress were found from April to October (Fig. 9, points 136–193). Using the annual cycle of the alongshore wind stress, the upwelling season was considered to last from April to October, and 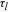 trends were calculated for these months (Fig. 10b). Negative trends were observed for almost the whole region except at the southernmost coast of Somalia (2–6°N) and the northernmost coast of Oman (18–22°N). Significant trends were observed mainly along the Somalia coast (6.5–10.5°N), with values between –1.5 10^−3^ Nm^–2^ dec^–1^ and –3.5 10^−3^ Nm^–2^ dec^–1^. Upwelling is induced by an alongshore current driven by the southwest monsoon in summer. With the onset of the northeast monsoon the circulation pattern reverses, causing a cessation of the upwelling. This region has been widely studied in terms of SST and biodiversity related to the occurrence of these upwelling events^61^,^62^,^63^,^64^,^65^. Nevertheless, few studies have focused on upwelling trends in terms of wind. Goes *et al.*^66^ reported an interannual escalation in the intensity of summer monsoonal winds accompanied by enhanced upwelling along the coast of Somalia (47–55°E, 5–10°N) using wind data from NCEP-NCAR reanalysis from 1997 to 2004. In contrast, using the same database and an advanced coupled atmosphere-ocean general circulation model, Izumo *et al.*^67^ detected a decrease in upwelling from 1979 to 2006 caused by anomalously weak southwesterly winds in late spring over the Arabian Sea. More recently, Piontkovski *et al.*^68^ described a declining trend in the zonal component of wind speed over the Sea of Oman (22–25°N) during summer monsoons from the late 1950s to 2010.

Results obtained for different upwelling systems around the world illustrate that it is not possible to describe a homogenous behaviour among them because trends change substantially, even in regions with similar oceanographic processes. Of the five major upwelling regions worldwide, increasing trends in upwelling were observed in the coastal areas of Benguela, Peru, Canary, and northern California, and the increases were statistically significant only for the two last systems. A general decrease in upwelling intensity was observed along the Chile, southern and central California, and central Somalia coasts, with significant values in all regions. Thus, no evidence for a general intensification of upwelling along these systems was observed.

The general trends found in our study were similar to those reported in studies in which wind stress data were used (Tables 1 and 2). It is important to note that controversial results were also obtained by different authors using the same variable and even the same period of time^2^,^15^, which indicates a dependence of results on the database used.

Different trends were also detected along the less studied upwelling regions. For example, significant decreasing trends were observed along the coast of Java and northern Kenya, whereas a tendency to an increase in upwelling-favourable winds was detected in western Australia. In the southern Caribbean upwelling region, a significant negative (positive) trend was found east (west) of 71.25°W.

Our analysis covered the last three decades (1982–2010), which is the recent period of strongest global warming, thus it allowed detailed analysis of the influence of warming processes on upwelling trends. In another study, Lima and Wethey^69^ estimated changes in coastal SST by exploring monthly warming patterns along the world’s coastline at a scale of 0.25° for the period 1982–2010. They found that even though most coastal areas worldwide have been warming, the magnitude of change has been highly heterogeneous in both space and season. They described a coastal SST decrease nearly year-round in the areas influenced by the California and Humboldt currents, which could be related to a tendency for intensification of upwelling following Bakun’s hypothesis. Nevertheless, our results revealed an increasing trend in upwelling along the northern California (38–42°N) and Peru coasts and negative trends along the Chile and southern-central California coasts (Table 2). On the other hand, Lima and Wethey^69^ found a general SST increase in the areas of the Canary, Benguela, and Somali currents, which could indicate a decrease of upwelled, cooler waters linked to an upwelling reduction. Our results only revealed a decrease in upwelling-favourable winds along the Somalia coast.

Among the upwelling systems analysed in the present study, Lima and Wethey^69^ found that coastal temperatures have been warming almost homogeneously throughout the year along the southern Caribbean upwelling region and along the Java and northern Kenya coasts. The western Australia coast has become colder from January to September. These results can be linked to the upwelling trends found in the present work. We detected decreasing trends in upwelling along the southern Caribbean, Java, and northern Kenya coasts, whereas a general upward trend was found along the western Australia coast.

These results delve into a possible discussion about whether the Bakun hypothesis is being fulfilled or not taking into account the present study and those mentioned in Tables 1 and 2. Even in regions where previously not many studies regarding upwelling trends existed, like the Java coast or the southern Caribbean upwelling region, different behaviours can be observed contradicting the general upward trend predicted by Bakun due to global warning.

## Author Contributions

Conceived and designed the experiments: R.V., I.A., F.S., M.dC. and M.G-G. Performed the experiments: R.V. Analyzed the data: R.V., I.A., F.S., M.dC. and M.G-G. Contributed reagents/materials/analysis tools: R.V., F.S. and M.G-G. Wrote the paper: R.V., I.A., M.dC. and M.G-G. All authors reviewed the manuscript.

## Additional Information

**How to cite this article**: Varela, R. *et al.* Has upwelling strengthened along worldwide coasts over 1982-2010? *Sci. Rep.* **5**, 10016; doi: 10.1038/srep10016 (2015).

## Figures and Tables

**Table 1:** Studies in which upwelling trends were analysed in terms of wind along the Benguela and Canary coasts.

**Zone**	**Author**	**Variable**	**Database (region)**	**Period (Upw. Season)**	**Trend**
**Benguela**	Patti *et al.* (2010)	WSt	COADS (30-20°S)	1958-2007 (Annual)	+
	Narayan *et al.* (2010)	WSt	COADS (36-26°S)	1960-2001 (Annual)	+
	Narayan *et al.* (2010)	WSt	NCEP/NCAR (36-26°S)	1960-2001 (Annual)	+
	Narayan *et al.* (2010)	WSt	ERA-40 (36-26°S)	1960-2001 (Annual)	0
	Pardo *et al.* (2011)	ET	NCEP/NCAR (32-20°S)	1970-2009 (Annual)	+
	Santos *et al.* (2012)	ET	NCEP/NCAR (30-16°S)	1970-2009 (Annual)	+
	**This work**	**WSt**	**CFSR** (30.1-23.3°S)	**1982-2010 (SEP-MAR)**	**+**
	**This work**	**WSt**	**CFSR** (20.8-16.4°S)	**1982-2010 (JUL-NOV)**	**+**
**Canary**	Cropper *et al.* (2014)	WSp	CFSR (11-35°N)	1981-2012 (JUN-AUG)	+
	Cropper *et al.* (2014)	WSp	NCEP/DOE II (11-35°N)	1981-2012 (JUN-AUG)	+
	Cropper *et al.* (2014)	WSp	ERA-Interim (11-35°N)	1981-2012 (JUN-AUG)	+
	Cropper *et al.* (2014)	WSp	20 Century (11-35°N)	1981-2012 (JUN-AUG)	+
	Cropper *et al.* (2014)	WSp	NASA-MERRA (11-35°N)	1981-2012 (JUN-AUG)	+
	McGregor *et al.* (2007)	WSt	COADS (35°N)	1950-1992 (Annual)	+
	Patti *et al.* (2010)	WSt	COADS (24-32°N)	1958-2007 (Annual)	+
	Narayan *et al.* (2010)	WSt	COADS (24-32°N)	1960-2001 (Annual)	+
	Narayan *et al.* (2010)	WSt	NCEP/NCAR (24-32°N)	1960-2001 (Annual)	−
	Narayan *et al.* (2010)	WSt	ERA-40 (24-32°N)	1960-2001 (Annual)	+
	Barton *et al.* (2013)	WSp	PFEL (15.5-41.5°N)	1967-2007 (Annual)	−
	Barton *et al.* (2013)	WSp	NCEP/NCAR (15-42.5°N)	1967-2007 (Annual)	−
	Barton *et al.* (2013)	WSp	ECMWF (15-40°N)	1967-2007 (Annual)	+
	Barton *et al.* (2013)	WSp	ICOADS (15-41°N)	1967-2007 (Annual)	+
	Barton *et al.* (2013)	WSp	WASWind (16-44°N)	1967-2007 (Annual)	−
	Gomez-Gesteira *et al.* (2008)	ET	PFEL (20-32°N)	1967-2006 (Monthly)	−
	Pardo *et al.* (2011)	ET	NCEP/NCAR (10-43°N)	1970-2009 (Annual)	−
	Santos *et al.* (2012)	ET	NCEP/NCAR (22-33°N)	1982-2010 (MAY-SEP)	+
	**This work**	**WSt**	**CFSR** (18.9-32.6°N)	**1982-2010 (APR-SEP)**	**+**

Variable abbreviations: WSt (Wind Stress), Ekman Transport (ET), WSp (Wind Speed).

**Table 2:** Studies in which upwelling trends were analysed in terms of wind along the Chile, Peru, and California coasts.

**Zone**	**Author**	**Variable**	**Database**	**Period (Upw. Season)**	**Trend**
**Chile**	Garreaud and Falvey (2009)	WSp	PRECIS (55-20°S)	1961-1990 (Annual)	0
	Goubanova *et al.* (2011)	WSp	IPSL-CM4 (40-0°S)	1970-1999 (Annual)	0
	Rahn and Garreaud (2013)	WSp	CFSR (36.4-30°S)	1979-2010 (Annual)	0
	Aravena *et al.* (2014)	ET	PFEL (34-29°S)	1980-2010 (Annual)	+
	**This work**	**WSt**	**CFSR** (37.6-28.9°S)	**1982-2010 (OCT-APR)**	**-**
**Peru**	Rahn and Garreaud (2013)	WSp	CFSR (15°)	1979-2010 (Annual)	+
	Gutierrez *et al.* (2011)	WSp	ERA-40 (14°S)	1958-2001 (Annual)	+
	Bakun *et al.* (2010)	WSt	COADS (10.5°S)	1948-2006 (Monthly)	+
	Goubanova *et al.* (2011)	WSp	IPSL-CM4 (15°S)	1970-1999 (Annual)	−
	Patti *et al.* (2010)	WSt	COADS (16-6°S)	1958-2007 (Annual)	+
	Narayan *et al.* (2010)	WSt	COADS (16-6°S)	1960-2001 (Annual)	+
	Narayan *et al.* (2010)	WSt	NCEP/NCAR (16-6°S)	1960-2001 (Annual)	−
	Narayan *et al.* (2010)	WSt	ERA-40 (16-6°S)	1960-2001 (Annual)	+
	Pardo *et al.* (2011)	ET	NCEP/NCAR (16-6.7°S)	1970-2009 (Annual)	−
	**This work**	**WSt**	**CFSR** (16.4-10.1°S)	**1982-2010 (MAY-OCT)**	**+**
**California**	Mendelssohn and Schwing (2002)	WSp	COADS (32-40°N)	1946-1990 (APR-SEP)	+
	Patti *et al.* (2010)	WSt	COADS (34-40°N)	1958-2007 (Annual)	+
	Narayan *et al.* (2010)	WSt	COADS (34-40°N)	1960-2001 (Annual)	+
	Narayan *et al.* (2010)	WSt	ERA-40 (34-40°N)	1960-2001 (Annual)	−
	Garcia-Reyes and Largier (2010)	WSp	NDBC (33-35°N)	1982-2008 (MAR-JUL)	−
	Garcia-Reyes and Largier (2010)	WSp	NDBC (35-42°N)	1982-2008 (MAR-JUL)	+
	Garcia-Reyes and Largier (2010)	ET	PFEL (34.5-42°N)	1982-2008 (MAR-JUL)	+
	Seo *et al.* (2012)	WSp	NDBC (33-35°N)	1980-2010 (JUN-AUG)	−
	Seo *et al.* (2012)	WSp	NDBC (35-42°N)	1980-2010 (JUN-AUG)	+
	Seo *et al.* (2012)	ET	CaRD10 (32-42°N)	1980-2010 (JUN-AUG)	+
	Rykaczewski and Checkley (2008)	ET	CaRD10 (34.5°N)	1948-2004 (Summer)	+
	Pardo *et al.* (2011)	ET	NCEP/NCAR (33-45°N)	1970-2009 (Annual)	0
	Iles *et al.* (2012)	ET	PFEL (33-45°N)	1967-2010 (Annual)	+
	**This work**	**WSt**	**CFSR** (33.9-36.1°N)	**1982-2010 (APR-SEP)**	−
	**This work**	**WSt**	**CFSR** (37.9-42.3°N)	**1982-2010 (APR-OCT)**	**+**

Variable abbreviations: WSt (Wind Stress), Ekman Transport (ET), WSp (Wind Speed).

## References

[b1] Bakun A. Global climate change and intensification of coastal upwelling. Science 247, 198–201 (1990). 17813287 10.1126/science.247.4939.198

[b2] Barton E. D., Field D. B., Roy C. Canary current upwelling: more or less? Prog. Oceanogr. 116, 167–178 (2013).

[b3] Sydeman W. J. *et al.* Climate change and wind intensification in coastal upwelling ecosystems. Science 345, 77 (2014). 24994651 10.1126/science.1251635

[b4] Saha S., *et al.* The NCEP Climate Forecast System Reanalysis. Bull. Amer. Meteorol. Soc. 91, 1015–1057 (2010).

[b5] García-Reyes M., Largier J. L., Sydeman W. J. Synoptic-scale upwelling indices and predictionsof phyto- and zooplankton populations. Prog. Oceanogr. 120, 177–188. (2014).

[b6] Bakun A. Coastal upwelling indices, west coast of North America, 1946-71. NOAA-NMFS, Technical Memorandum, pp. 1–13 (1973).

[b7] Mason J. E., Bakun A. Upwelling index update, U.S. west coast 33N-48N latitude. NOAA-NMFS, Southwest Fisheries Center, Tech. Memo 67, 8 1 pp (1986).

[b8] Cury P., Roy C. Optimal environmental window and pelagic fish recruitment success in upwelling areas. Can. J. Fish. Aquat. Sci. 46, 670–680 (1989).

[b9] Santos F., Gomez Gesteira M., deCastro M., Alvarez I. Differences in coastal and oceanic SST trends due to the strengthening of coastal upwelling along the Benguela current system. Contin. Shelf Res. 34, 79–86 (2012).

[b10] Santos F., deCastro M., Gómez-Gesteira M., Álvarez I. Differences in coastal and oceanic SST warming rates along the Canary upwelling ecosystem from 1982 to 2010. Cont. Shelf Res. 47, 1–6. (2012).

[b11] Alvarez I., Gómez-Gesteira M., deCastro M., Carvalho D. Comparison of different wind products and buoy wind data with seasonality and interannual climate variability in the southern Bay of Biscay (2000–2009). Deep-Sea Res. Pt II. 106, 38–48. (2014).

[b12] Carvalho D., Rocha A., Gómez-Gesteira M., Silva Santos C. Comparison of reanalyzed, analyzed, satellite-retrieved and NWP modelled winds with buoy data along the Iberian Peninsula coast. Remote Sens. Environ. 152, 480–492. (2014).

[b13] Carvalho D., Rocha A., Gómez-Gesteira M. Offshore wind energy resource simulation forced by different reanalyses: comparison with observed data in the Iberian Peninsula. Appl. Energy. 134, 57–64. (2014).

[b14] Patti B. *et al.* Effect of atmospheric CO2 and solar activity on wind regime and water column stability in the major global upwelling areas. Est. Coast. Shelf Sci. 88, 45–52 (2010).

[b15] Narayan N., Paul A., Mulitza S., Schulz M. Trends in coastal upwelling intensity during the late 20th century. Ocean Sci. 6, 815–823 (2010).

[b16] Pardo P., Padín X., Gilcoto M., Farina-Busto L., Pérez F. Evolution of upwelling systems coupled to the long term variability in sea Surface temperatura and Ekman transport. Clim. Res. 48, 231–246 (2011).

[b17] Cropper T. E., Hanna E., Bigg G. R. Spatial and temporal seasonal trends in coastal upwelling off Northwest Africa, 1981–2012. Deep-Sea Res. 1, 94–111 (2014).

[b18] McGregor H. V., Dima M., Fischer H. W., Mulitza S. Rapid 20th-Century Increase in Coastal Upwelling off Northwest Africa. Science ; 315, 637–639 (2007). 17272719 10.1126/science.1134839

[b19] Gómez-Gesteira M. *et al.* Spatio-temporal upwelling trends along the Canary Upwelling System (1967−2006). In: Trends and directions in climate research. Ann. N. Y. Acad. Sci. 1146, 320−337 (2008). 19076422 10.1196/annals.1446.004

[b20] Santos F., Gomez-Gesteira M., deCastro M., Alvarez I. Upwelling along the western coast of the Iberian Peninsula: dependence of trends on fitting strategy. Clim. Res. 48, 213–218 (2011).

[b21] Black D. E. *et al.* Eight centuries of North Atlantic Ocean atmosphere variability, Science, 286, 1709–1713 (1999). 10576732 10.1126/science.286.5445.1709

[b22] Muller-Karger F. *et al.* Processes of coastal upwelling and carbon flux in Cariaco Basin, Deep Sea Res., Part II, 51, 927–943 (2004).

[b23] Andrade C. A., Barton E. D. The Guajira upwelling system, Cont. Shelf Res., 25, 1003–1022 (2005).

[b24] Ruiz-Ochoa M., Beier. E., Bernal G., Barton E. D. Sea surface temperature variability in the Colombian Basin, Caribbean Sea, Deep Sea Res., Part I, 64, 43–53, (2012).

[b25] Rueda-Roa D.T., Muller-Karger F.E. The southern Caribbean upwelling system: sea surface temperature, wind forcing and chlorophyll concentration patterns. Deep Sea Res. Part I Oceanogr Res. Pap 78, 102–114. (2013).

[b26] Garreaud R. D., Falvey M. The coastal winds off western subtropical South America in future climate scenarios. Int. J. Climatol., 29, 543–554 (2009).

[b27] Goubanova K., *et al.* Statistical downscaling of sea-surface wind over the Peru-Chile upwelling region: Diagnosing the impact of climate change from the IPSL-CM4 model, Clim. Dyn. 36, 1365–1378 (2011).

[b28] Rahn D. A., Garreaud R. D. A synoptic climatology of the near-surface wind along the west coast of South America. Int. J. Climatol. 34, 780–792 (2013).

[b29] Aravena G., Broitman B., Stenseth N. C. Twelve years of change in coastal upwelling along the central-northern coast of Chile: Spatially heterogeneous responses to climatic variability. PLoS ONE 9, e90276, 10.1371/journal.pone.0090276 (2014). 24587310 PMC3938675

[b30] Gutiérrez D., *et al.* Coastal cooling and increased productivity in the main upwelling zone off Peru since the mid-twentieth century. Geophys. Res. Lett., 38, 6. (2011).

[b31] Bakun A., Field D., Redondo-Rodriguez A., Weeks S. Greenhouse gas, upwelling-favorable winds, and the future of coastal ocean upwelling ecosystems. Glob. Change Biol. 16, 1213–28 (2010).

[b32] Mendelssohn R., Schwing F. B. Common and uncommon trends in SST and wind stress in the California and Peru-Chile current systems, Prog. Oceanogr, 53, 141–162, (2002).

[b33] Garcia-Reyes M., Largier J. Observations of increased wind-driven coastal upwelling off central California. J. Geohys. Res., C, 115, C04010, 10.1029/2009JC005576 (2010).

[b34] Seo H., Brink K. H., Dorman C. E., Koracin D., Edwards C. A. What determines the spatial pattern in summer upwelling trends on the U.S. West Coast? J. Geophys. Res. 117, C08012, (2012).

[b35] Rykaczewski R. R., Checkley D. M. Influence of ocean winds on the pelagic ecosystem in upwelling regions, Proc. Natl. Acad. Sci., 105, 1965–1970, (2008). 18250305 10.1073/pnas.0711777105 PMC2538866

[b36] Iles A. C., *et al.* Climate-driven trends and ecological implications of event-scale upwelling in the California Current System. Glob. Change Biol. 18, 783−796 (2012)

[b37] Church J. A., Cresswell G., Godfrey J. S. The Leeuwin Current. In: Neshyba S.J., Mooers Ch. N. K., Smith R.L., Barber R.T., editors . Poleward flow along eastern ocean boundaries. Coast. Estuar. Stud. 34. New York: Springer-Verlag. p 230–54 (1989).

[b38] Smith R. L., Huyer A., Godfrey J. S., Church J. A. The Leeuwin current off western Australia, 1986–1987, J. Phys. Oceanogr., 21, 323–345, (1991).

[b39] Pearce A. F. Eastern boundary currents of the southern hemisphere. J. R. Soc. W. Aust. 74, 35–45 (1991).

[b40] Rousseaux C. S. G., Lowe R. J., Feng M., Waite A. M., Thompson P. A. The role of the Leeuwin Current and mixed layer depth on the autumn phytoplankton bloom off Ningaloo Reef, Western Australia. Cont. Shelf Res. 32, 22–35, (2012).

[b41] Gersbach G. H., Pattiaratchi C. B., Ivey G. N., Cresswell G. R. Upwelling on the south-west coast of Australia — source of the Capes Current? Cont. Shelf Res. 19, 363–400 (1999).

[b42] Wilson S. G., Carleton J. H., Meekan M. G. Spatial and temporal patterns in the distribution and abundance of macrozooplankton on the southern North West Shelf, Western Australia. Est. Coast. Shelf Sci. 56, 897–908 (2003).

[b43] Hanson C. E., Pattiaratchi C. B., Waite A. M. Sporadic upwelling on a downwelling coast: phytoplankton responses to spatially variable nutrient dynamics off the Gascoyne region of Western Australia. Cont. Shelf Res. 25, 1561–1582 (2005).

[b44] Woo M., Pattiaratchi C. B., Schroeder W. Summer surface circulation along the Gascoyne continental shelf, western Australia. Cont. Shelf Res. 26, 132–152 (2006).

[b45] Furnas M. Intra-seasonal and inter-annual variations in phytoplankton biomass, primary production and bacterial production at North West Cape, Western Australia: Links to the 1997–1998 El Niño event. Cont. Shelf Res. 27, 958–980 (2007).

[b46] Thompson P. A., *et al.* Contrasting oceanographic conditions and phytoplankton communities on the east and west coasts of Australia. Deep-Sea Res. 58, 645–663 (2011).

[b47] Xu J. *et al.* Dynamics of the summer shelf circulation and transient upwelling off Ningaloo Reef Western Australia, J. Geophys. Res., 118, 1099–1125, (2013).

[b48] Susanto R. D., Gordon A. L., Zheng Q. Upwelling along the coasts of Java and Sumatra and its relation to ENSO. Geophys. Res. Lett., 28, 1599–1602 (2001).

[b49] Gordon A. L. Oceanography of the Indonesian seas and their throughflow, Oceanography, 18, 14–27 (2005).

[b50] Qu T., Du Y., Strachan J., Meyers G., Slingo J. M. Sea surface temperature and its variability in the Indonesian region, Oceanography Wash. D. C., 18, 50–62 (2005).

[b51] Susanto R. D., Marra J. Effect of the 1997/98 El Niño on chlorophyll a variability along the coasts of Java and Sumatra. Prog. Oceanogr., 18, 124–127 (2005).

[b52] Andruleit H. Status of the Java upwelling area (Indian Ocean) during the oligotrophic northern hemisphere winter monsoon season as revealed by coccolithophores. Mar. Micropaleontol. 64, 36–51. (2007).

[b53] Baumgart A., Jennerjahn T., Mohtadi M., Hebbeln D. Distribution and burial of organic carbon in sediments from the Indian Ocean upwelling region off Java and Sumatra, Indonesia. Deep-Sea Res. 57, 458–467 (2010).

[b54] McClanahan T. R. Seasonality in East Africa’s coastal waters. Mar. Ecol. Prog. Ser. 44, 191–199 (1988).

[b55] Semeneh M. F., Dehairs F., Goeyens L. Uptake of nitrogenous nutrients by phytoplankton in the tropical Western Indian Ocean (Kenyan Coast): monsoonal and spatial variability. In: Heip C. M. A., Hemminga M. J. M. (Eds) , Monsoons and Ecosystems in Kenya. Kenya Mar. Fish. Res. Inst., Mombasa, Kenya: 101–104 (1995).

[b56] Duineveld G. C. A., *et al.* Benthic respiration and standing stock on two contrasting continental margins in the western Indian Ocean: The Yemen-Somali upwelling region and the margin off Kenya, Deep Sea Res., Part II, 44, 1293–1317 (1997).

[b57] Kromkamp J., *et al.* Primary production by phytoplankton along the Kenyan coast during the SE monsoon and November intermonsoon 1992, and the occurrence of Trichodesmium, Deep Sea Res. Part II, 44, 1195–1212 (1997).

[b58] Mengesha S., Dehairs F., Elskens M., Goeyens L. Phytoplankton nitrogen nutrition in the western Indian Ocean: Ecophysiological adaptations of neritic and oceanic assemblages to ammonium supply, Est. Coast. Shelf Sci., 48, 589–598 (1999).

[b59] Mwaluma J., Osore M., Kamau J., Wawiye P. Composition, abundance and seasonality of zooplanktonin Mida Creek, Kenya. Western Indian Ocean. J. Mar. Sci. 2, 147–155 (2003).

[b60] Muthumbi A. W., Vanreusel A., Duineveld G., Soetaert K., Vincx M. Nematode community structure along the continental slope off the Kenyan Coast, Western Indian Ocean. Int. Rev. Gesamten Hydrobiol., 89, 188–205 (2004).

[b61] Wyrtki K. Physical oceanography of the Indian Ocean. Pp. 18–36 in B. Zeitzschel, ed. The biology of the Indian Ocean. Springer-Verlag, New York (1973).

[b62] Prell W. L. Variation of monsoonal upwelling: a response to changing solar radiation. In: Hansen J., T. Takahashi T. (Eds.) , Clim. Proc. and Clim. Sens., AGU, pp. 48–57 (1984).

[b63] Koning E., *et al.* Selective preservation of upwelling-indicating diatoms in sediments off Somalia, NW Indian Ocean. *Deep-Sea Res.* I 48, 2473–2495 (2001).

[b64] de Boyer Montegut C., *et al.* Simulated seasonal and interanual variability of mixed layer heat budget in the northern Indian Ocean, J. Clim., 20, 3249– 3268 (2007).

[b65] Valsala K. V. Different spreading of Somali and Arabian coastal upwelled waters in the northern Indian Ocean: A case study, J. Oceanogr., 65, 803–816 (2009).

[b66] Goes J. I., Thoppil P. G., Gomes H. do R., Fasullo J. T. Warming of the Eurasian landmass is making the Arabian Sea more productive. Science, 308, 545–547. (2005). 15845852 10.1126/science.1106610

[b67] Izumo T., *et al.* The role of the western Arabian Sea upwelling in Indian monsoon rainfall variability, J. Clim., 21, 5603–5623 (2008).

[b68] Piontkovski S. A., AL-Gheilani H. M. H., Jupp B. P., Al-Azri A. R., Al-Hashmi K. A. Interannual changes in the Sea of Oman ecosystem. Open Mar. Biol. J. 6, 38–52 (2012).

[b69] Lima F. P., Wethey D. S. Three decades of high-resolution coastal sea surface temperatures reveal more than warming. Nature Commun. 3, 704 (2012). 22426225 10.1038/ncomms1713

